# AGEs/RAGE blockade downregulates Endothenin-1 (ET-1), mitigating Human Umbilical Vein Endothelial Cells (HUVEC) injury in deep vein thrombosis (DVT)

**DOI:** 10.1080/21655979.2021.1917980

**Published:** 2021-04-25

**Authors:** Yunxin Zhang, Jianlong Liu, Wei Jia, Xuan Tian, Peng Jiang, Zhiyuan Cheng, Jinyong Li

**Affiliations:** Department of Vascular Surgery, 1-7 Beijing Jishuitan Hospital, Beijing, China

**Keywords:** AGE/rage, ET-1, HSA, DVT, HUVECS

## Abstract

This study is aimed at identifying the roles of AGE/RAGE and ET-1 in deep vein thrombosis (DVT). Advanced glycation end products (AGEs) in glycated human serum albumin (M-HSA) were detected by ELISA. The viability of HUVECs was examined by CCK-8 assay. Flow cytometry was performed to detect cell apoptosis, followed by ELISA for the detection of inflammatory cytokine level and oxidative stress level in HUVECs. Immunofluorescence was performed to detect ET-1 and eNOS expression. The expression of specific proteins was assayed by western blot. As a result, decreased HUVEC viability was observed after stimulation with M-HSA, whereas RAGE inhibitor improved it. Cell apoptosis showed the opposite trend. Additionally, M-HSA-induced inflammatory cytokine release and oxidative stress of HUVECs were both alleviated by RAGE inhibitor. RAGE inhibitor also increased the levels of NO and eNOS while decreasing the level of ET-1 in M-HSA-stimulated HUVECs. Furthermore, decreased protein expression of Bax, cleaved-caspase3, RAGE, p65, ET-1 and iNOS was observed after treatment with RAGE inhibitor, in addition to increased protein expression of Bcl-2 and eNOS. In conclusion, blocking AGE/RAGE pathway downregulates ET-1, thereby mitigating HUVEC damage in DVT.

## GRAPIC ABSTRACT

**Figure:**
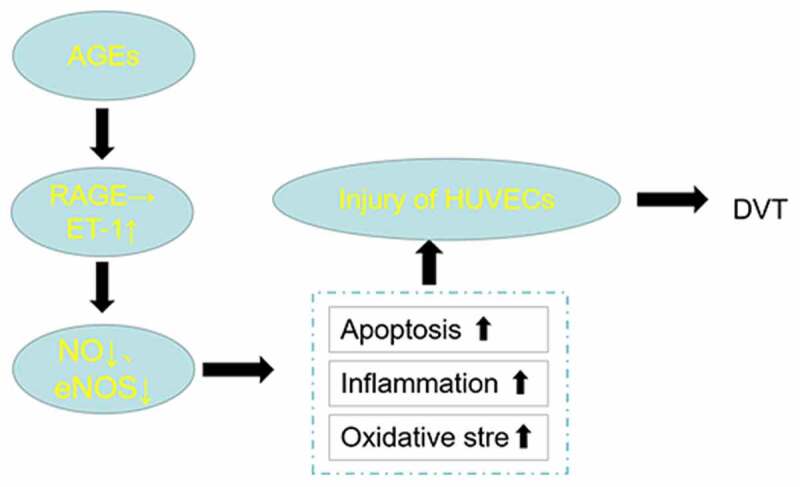

## Introduction

The formation of deep vein thrombosis (DVT) is a result from the combined action of slow blood flow, increased blood thickness and damage to the inner lining of the vein [1,2]. Age, blood glucose, blood pressure, blood lipid and tumor are common influential factors for the occurrence of DVT [3]. Additionally, patients after orthopedic surgery are also predisposed to DVT [4]. Except for a few cases where the thrombosis is dissolved spontaneously or confined to the site of occurrence, in most cases the thrombosis will spread to the whole lower limb. If not diagnosed and treated in time, DVT can seriously impact the quality of life of patients as they may develop pulmonary embolism [5]. In particular, endothelial cell injury and dysfunction are crucial factors in the development of DVT. Prior studies have reported that excessive oxidative stress is a common cause of vascular endothelial cell injury [6,7]. Additionally, intracellular reactive oxygen species (ROS) can exacerbate apoptosis in vascular endothelial cells and decrease the expression of anti-apoptotic molecules [8]. However, the specific molecular mechanisms involved in inducing endothelial cell injury are still unclear.

Endothelin-1 (ET-1), an endogenous peptide with significant vasoconstrictive activity, was reported to be associated with inflammation inducement and contribute to endothelial injury [9]. Some studies have illustrated significantly higher ET-1 level in thrombosis model compared to the control group, revealing that ET-1 may serve as a marker for DVT diagnosis [10,11].

Advanced glycation end products (AGEs) and their receptors (RAGE) were first studied in diabetes. AGEs have proven to be overproduced in the elderly [12], especially those with diabetes [13], hypertension [14] and/or atherosclerosis [15]. And it was found that inhibiting the synthesis of AGEs and reducing the overexpression of RAGE were the main ways to inhibit glycation of proteins [16]. Studies have corroborated that RAGE pathway is associated with neuroinflammation, oxidative stress and the occurrence of many diseases such as insulin resistance and diabetic nephropathy [17–19]. Furthermore, ET-1 expression closely correlates with the level of AGEs and is regulated by the RAGE signaling pathway in mouse model of diabetic mellitus erectile dysfunction [20]. Endothelial dysfunction predisposes to DVT and study demonstrates that glycine may inhibit the AGE/RAGE pathway and subsequent oxidative stress by improving Glo1 function [21]. Based on these findings, we conducted this study to identify how AGE/RAGE is involved in the development of reducing human umbilical vein endothelial cells (HUVECs) injury and whether blocking AGEs/RAGE axis downregulates the level of ET-1 and thereby reduces HUVECs injury in DVT.

## Materials and methods

### Cell culture and treatment

Primary Human Umbilical Vein Endothelial Cells (HUVECs) were purchased from ThermoFisher Scientific (Waltham, MA, USA) and were cultured in Medium 200 (ThermoFisher; Cat. No. M-200-500) supplemented with 1% penicillin/streptomycin in a humidified incubator with 5% CO_2_ and 95% air at 37°C. The medium was renewed after 36 h of cell culture and was then changed every other day until 80% confluency. RAGE inhibitor FPS-ZM1 was sourced from MedChem Express (Monmouth Junction, NJ, USA).

## Preparation of glycated human serum albumin

The preparation of glycated human serum albumin was based on previous studies [22,23]. Briefly speaking, 15 mg/ml HSA was incubated with 1 mM of 3-deoxyglucosone (3-DG) for 14 days at 37°C to modify the HSA (M-HSA). AGEs including NƐ-carboxy methyl lysine (CML) and imidazolone in M-HSA were detected by ELISA.

## Enzyme-Linked Immune Sorbent Assay (ELISA)

ELISA kit (Yuanmu, Shanghai, China) was used for the detection of AGEs contained in M-HSA as well as the level of pro-inflammatory cytokines. 100 μl supernatant of the samples was added to each well of a 96-well plate and incubated for 60 min at room temperature with added 100 μl biotin antibody. The samples were then incubated with 100 μl/well of Streptavidin labeled with horseradish peroxidase at room temperature in the dark for 20 min. Next, chromogen TMB solution 100 μl/well was added for 15–20 min of incubation. The assay was ended by addition of 50 μl termination solution to each well.

## Cell Counting Kit-8 Assay (CCK-8)

CCK-8 (Beyotime, Nanjing, China) assay was performed to determine the viability of M-HSA-stimulated HUVECs. HUVECs were seeded into a 96-well plate at a density of 2 × 10^3^ cells/well. After addition of 10 μl CCK-8 solution to each well, the cells were continuously incubated for 1 h. Cell viability was detected by measuring the absorbance at 450 nm wavelength with a microplate reader (ThermoFisher Scientific Waltham, MA, USA).

## Flow cytometry

Flow cytometry was performed to detect HUVEC apoptosis. In the short term, HUVECs were first incubated with Hoechst 33,342/bisbenzimide H33342 (Beyotime, Nanjing, China) at 37°C for 10 min. The samples were then centrifuged at 500–1000 r/min for 5 min at low temperature to discard the staining solution. After addition of 1.0 mL Propidium Iodide (PI) solution to stain the cells at 4°C in the dark for 15 min, the samples were filtrated 1 time with a Falcon® strainer (Corning Incorporated, Corning, NY, USA). Finally. CytoFLEX flow cytometer (Beckman Coulter, Miami, FL, USA) was utilized for cell apoptosis analysis. The sum of apoptosis rate in right upper quadrant (Q2, late apoptotic cells) and right lower quadrant (Q3, early apoptotic cells) were considered as the cell apoptosis rate.

## Oxidative stress level detection

Reactive Oxygen species (ROS) Assay Kit (Beyotime, Nanjing, China) was used for the detection of ROS release in HUVECs in different groups. Lipid Peroxidation Malondialdehyde (MDA) Assay Kit (Beyotime, Nanjing, China) detected MDA content. Superoxide Dismutase (SOD) Assay Kit (Beyotime, Nanjing, China) determined the activity of SOD. All three assays were performed in accordance with the guidelines provided by the manufacturer.

## Nitric Oxide (NO) detection

NO is implicated in various physiological and pathological processes in human body. We thus detected the level of NO in HUVECs using Total Nitric Oxide Assay Kit/Nitrate Assay Kit (Beyotime, Nanjing, China). Cells were first lysed with Cell and Tissue Lysis Buffer for Nitric Oxide Assay (S3090) before successive addition of Griess Reagent I and II included in the kit. Absorbance was measured at 540 nm wavelength.

## Immunofluorescence

The expressions of ET-1 and endothelial nitric oxide synthase (eNOS) in HUVECs were detected by Immunol Fluorescence Staining Kit (Elabscience, Wuhan, China). The cells were collected and fixed with 0.5 mL fixing solution for 10 min. After final centrifugation, the slide was sealed with mounting solution for 60 min. The cells were then incubated with diluted primary antibody overnight at 4°C before incubation with fluorescently labeled secondary antibody in darkness for 60 min. Lastly, the fluorescence was observed and graphed with a fluorescence microscope (Leica, Wetzlar, Germany).

## Western blot

Total protein was extracted by RIPA lysis buffer with 1 mM PMSF (Beyotime, Nanjing, China). Protein concentration was determined by BCA kit (Beyotime, Nanjing, China). SDS-PAGE (ThermoFisher, Waltham, MA, USA) was added to the samples for boiling for 5 min to obtain denatured protein. Electrophoresis was then performed for 90 min and stopped when the protein was properly separated. PVDF membrane (Corning Incorporated, Corning, NY, USA) with transferred protein was blocked by nonfat milk for 10 min on a shaking bed at room temperature. After incubation with primary antibody overnight at 4°C, the cells were incubated with secondary antibody for 1 h at room temperature. The membrane was washed 3 times with TBST (Aladdin, Shanghai, China) at last for 5 min each time before protein detection by TMB Chromogen Solution (Beyotime, Nanjing, China). GAPDH was used as the control.

## Statistical analysis

All experiments were repeated threefold and the data are presented as mean ± standard deviation (SD). GraphPad Prism 6 was used for data analysis. One-way ANOVA followed by student’s t-test was applied to group comparisons. P value less than 0.05 stands for statistical significance.

## Results

This study focused on the mechanism of the effect of AGEs and RAGE on HUVEC injury. By preparing M-HSA containing AGEs for intervention, the activation of AGEs/RAGE on HUVEC injury – an important factor contributing to DVT – was verified, consistent with previous relevant clinical observations. Further studies next revealed that ET-1 was activated during cellular injury by AGEs/RAGE, and the experimental results suggested that the oxidative stress and inflammatory response of HUVEC cells were significantly elevated, possibly by affecting the NOS synthesis pathway, thereby causing cellular injury. Next, artificial down-regulation of ET-1 expression level could partially reverse the HUVEC damage caused by activation of AGEs/RAGE, NOS synthesis and OS and inflammatory response levels, further demonstrating the role and potential mechanism of ET-1 in this process

## Increased production of AGEs in M-HSA

**Figure 1.**
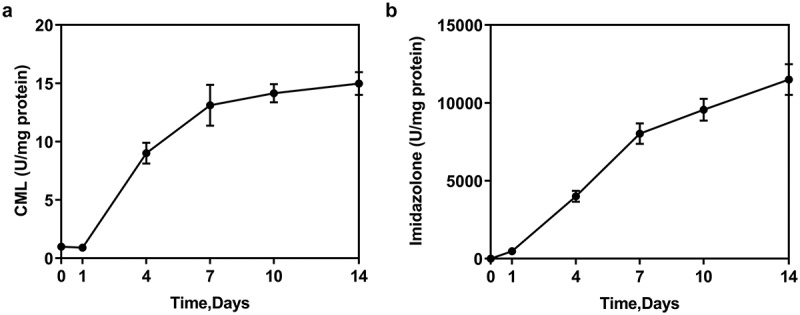
Concentration of AGEs (CML and imidazolone) in M-HSA (ELISA)

Two types of AGEs, CML and imidazolone in M-HSA were detected by ELISA, the result of which showed increased CML and imidazolone levels as the days went on (Figure 1). AGEs were thus successfully induced in our study.

## AGEs/RAGE inhibition increases the viability of M-HSA-stimulated HUVECs

**Figure 2.**
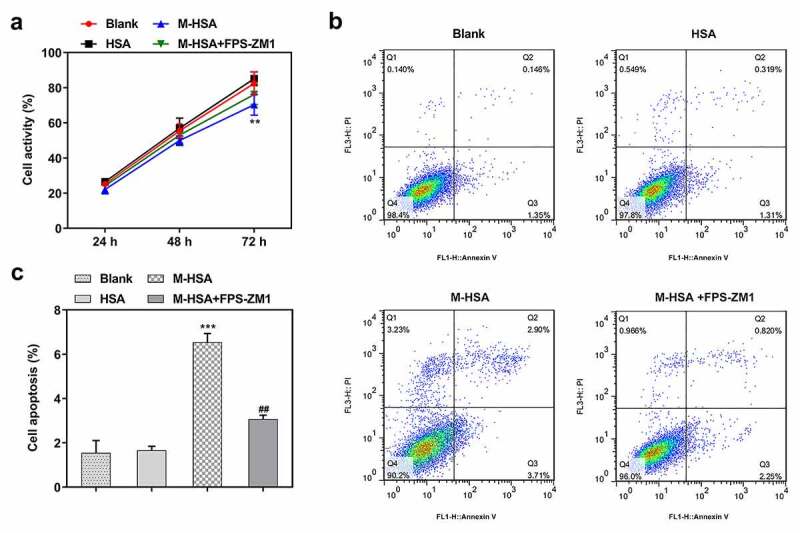
**A** HUVEC viability in the blank group, HSA, M-HSA and M-HSA+FPS-ZM1 (CCK-8) **P < 0.01; **B-C** HUVEC viability in the blank group, HSA, M-HSA and M-HSA+FPS-ZM1 (flow cytometry) ^##^P < 0.01, ***P < 0.001

To identify the role of AGEs/RAGE in DVT, we first compared the viability of M-HSA-stimulated HUVECs before and after RAGE inhibition. It was found that treatment with FPS-ZM1 increased the viability of M-HSA-stimulated HUVECs, in comparison to the M-HSA group (Figure 2a). Moreover, flow cytometry detected elevated apoptosis level of HUVECs following M-HSA stimulation, which was significantly decreased by FPS-ZM1 (-c). These results suggest that AGEs/RAGE inhibition increases the viability and decreases the apoptosis of M-HSA-stimulated HUVECs.

## AGEs/RAGE inhibition alleviates M-HSA-induced inflammation and oxidative stress in HUVECs

**Figure 3.**
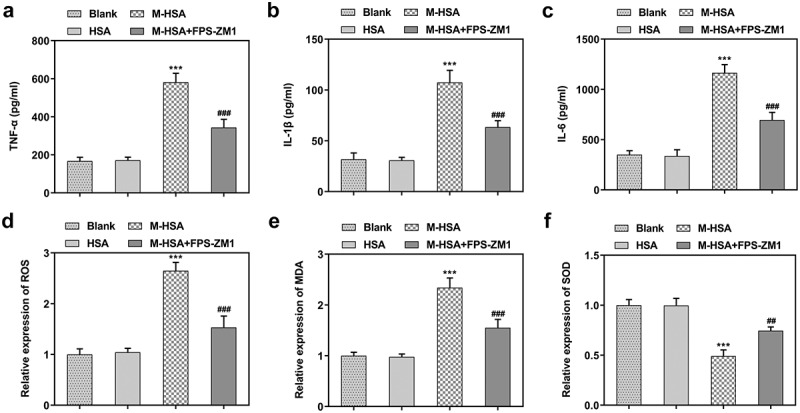
**A-C** relative expressions of pro-inflammatory cytokines in the blank group, HSA, M-HSA and M-HSA+FPS-ZM1 (ELISA) ***P < 0.001, ^###^P < 0.001; **D-E** relative expressions of ROS, MDA and SOD in the blank group, HSA, M-HSA or M-HSA+FPS-ZM1 (commercial kits) ^##^P < 0.01 ***P < 0.001, ^###^P < 0.001

Pro-inflammatory cytokines and oxidative stress level were detected to find out how AGEs/RAGE affects inflammatory response and oxidative stress in DVT. We noticed conspicuously increased release of TNF-α, IL-1β and IL-6 after HUVECs were stimulated with M-HSA (-c). However, the release of pro-inflammatory cytokines was suppressed to a great extent after treatment with RAGE inhibitor. In addition, it was found that the relative expression of ROS and MDA showed marked increase in HUVECs following M-HSA stimulation, while they were downregulated after RAGE inhibition (-e). Meanwhile, the relative expression of SOD was greatly downregulated in HUVECs after M-HSA stimulation, which was then upregulated after FPS-ZM1 treatment (figure 3f). Collectively, the above results indicate that blocking AGEs/RAGE can alleviate M-HSA-induced inflammation and oxidative stress in HUVECs.

## AGEs/RAGE inhibition increases NO and ET-1 levels and decreases eNOS level in M-HSA-stimulated HUVECs

**Figure 4.**
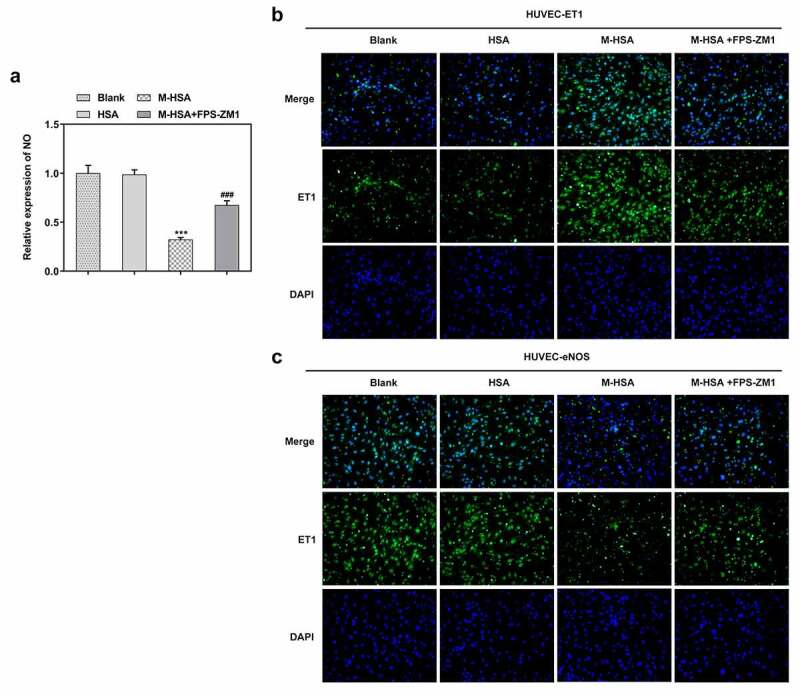
**A** relative expression of NO in the blank group, HSA, M-HSA or M-HSA+FPS-ZM1 (commercial kit) ***P < 0.001, ^###^P < 0.001; **B-C** Images of CE-1 and eNOS expressions in the blank group, HSA, M-HSA and M-HSA+FPS-ZM1 (commercial kit)

Vasodilatory NO is an unneglectable factor when it comes to examining the condition of HUVECs. We detected the level of NO in HUVECs in different groups and found that it was significantly decreased by M-HSA stimulation, whereas FPS-ZM1 increased it noticeably (Figure 4a). -c demonstrated the results of IF assay, where the level of ET-1 increased and that of eNOS decreased in M-HSA-stimulated HUVECs, while FPS-ZM1 reversed their expression. These results suggest that blocking AGEs/RAGE signaling can improve NO production, upregulation ET-1 level while decreasing eNOS expression in M-HSA-stimulated HUVECs.

## Verified effects of AGEs/RAGE blockade on M-HSA-stimulated HUVECs

**Figure 5.**
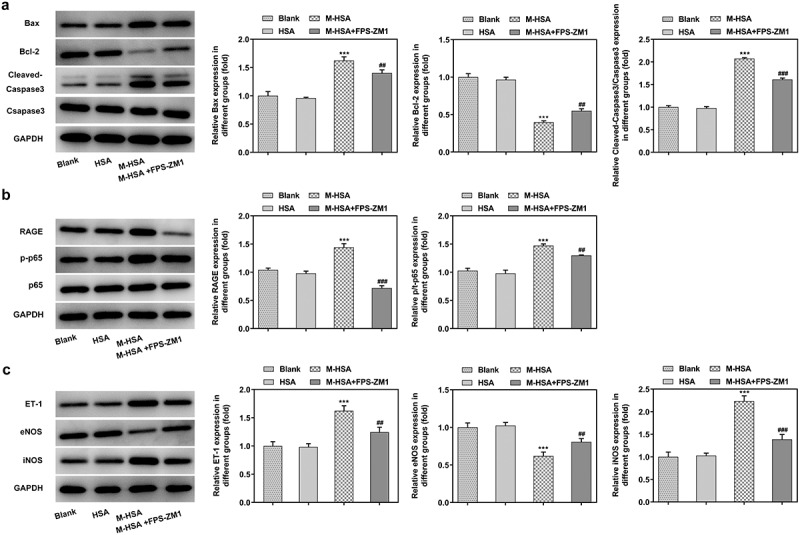
**A** relative expressions of Bax, Bcl-2 and C-Caspase3 in the blank group, HSA, M-HSA and M-HSA+FPS-ZM1 (WB) ***P < 0.001, ^##^P < 0.01, ^###^P < 0.001; **B** relative expressions of RAGE and p-p65 in the blank group, HSA, M-HSA and M-HSA+FPS-ZM1 (WB) ***P < 0.001, ^##^P < 0.01, ^###^P < 0.001; **C** relative expressions of ET-1, eNOS and iNOS in the blank group, HSA, M-HSA or M-HSA+FPS-ZM1 (WB) ***P < 0.001, ^##^P < 0.01, ^###^P < 0.001

Western blot analysis was performed to verify the above experimental results. First of all, the expression of pro-apoptotic Bax and cleaved caspase3 was decreased while the expression of anti-apoptotic Bcl-2 was increased by inhibiting RAGE in M-HSA-stimulated HUVECs (Figure 5a). Additionally, the expression of RAGE and p65 was downregulated by RAGE inhibitor (Figure 5b). Lastly, the protein expression of ET-1 and inducible nitric oxide synthase (iNOS) was found to be reduced after RAGE inhibition, whereas eNOS expression exhibited significant elevation (Figure 5c). These results further verify the effects of inhibiting AGEs/RAGE on M-HSA-stimulated HUVECs.

## Discussion

Deep vein thrombosis (DVT) refers to an abnormal clotting of blood in deep veins. It is a disorder of venous return in lower limbs and is accompanied by a series of clinical manifestations such as limb swelling, leg pain and secondary varicose veins [24]. Although symptoms may not always appear, DVT increases the risk of pelvic and lower extremity fracture and even brain injury [25]. Therefore, it is necessary to prevent or treat DVT effectively. The occurrence and development have a proven association with inflammatory response [26], reactive oxygen species [27] and expanded blood vessels [28].

The AGEs/RAGE axis has been substantiated to be closely linked to inflammation, oxidative stress and a variety of diseases including diabetes and cardiovascular diseases [19,29,30]. A recent report has shown that RAGE deletion can potentially prevent chronic kidney disease-induced platelet hyperactivation and arterial thrombosis [31]. In our study, we observed weakened HUVECs viability and increased cell apoptosis after M-HSA stimulation and largely restored level of those after treatment with RAGE inhibitor FPS-ZM1. Numerous studies have shown that inflammation is closely related to blood coagulation and plays a modulatory role in the pathophysiology of deep vein thrombosis [32,33]. Our results demonstrated promoted release of pro-inflammatory cytokines IL-6, IL-1β and TNF-α in M-HSA-stimulated HUVECs, which was suppressed after RAGE inhibition. Furthermore, overproduction of reactive oxygen species (ROS) in red blood cells can activate thrombotic events, which implies the association between oxidative stress and DVT [34,35]. In our study, RAGE inhibition greatly suppressed the levels of MDA and ROS and elevated the level of anti-oxidant SOD in M-HSA-stimulated HUVECs, effectively alleviated AGEs-induced oxidative stress. Additionally, we also also detected the level of nitric oxide (NO), and signaling molecule with anti-inflammatory and vasodilatory qualities that helps preventing the occurrence of thrombosis [36,37]. Our study demonstrated decreased NO level in M-HSA-stimulated HUVECs, whereas inhibiting RAGE increased its level significantly.

ET-1, an endogenous polypeptide discovered around 30 years ago, is an essential participant in vasoconstriction and mitogenesis [38]. Studies have shown that ET-1 may be involved in retinal vein occlusion and the formation of micro thrombosis [39,40]. Increased expression of ET-1 was also described as a thrombosis-related factor in a study on DVT [7]. A study has found that there exists a positive correlation between AGEs levels and ET-1 production in women with polycystic ovary syndrome (PCOS) [41]. According to another report, AGEs can regulate the expression of ET-1 in the body through RAGE signaling pathway [42]. Additionally, ET-1-induced vasoconstriction is exacerbated by the increase of AGEs in type 2 diabetes mellitus [43]. Consistently, highly expressed ET-1 was observed M-HSA-stimulated HUVECs in our study, whereas RAGE inhibitor notably suppressed ET-1 expression level.

## Conclusion

In our *in vitro* model of DVT, AGEs/RAGE blockade alleviates glycated HSA-induced injury of HUVECs by downregulating ET-1. This study is significant in that it not only describes the underlying mechanism of AGEs/RAGE axis in DVT, but also provides a novel approach to the prevention, diagnosis and treatment of DVT.

## Research highlights

1. AGEs production was increased in M-HSA.

2. AGEs/RAGE inhibition increases the viability of M-HSA-stimulated HUVECs

3. AGEs/RAGE inhibition alleviates M-HSA-induced inflammation and oxidative stress in HUVECs.

4. AGEs/RAGE inhibition increased NO and ET-1 levels and decreased eNOS levels in M-HSA-stimulated HUVECs.

5. AGEs/RAGE blockade alleviated glycosylated HSA-induced injury in HUVECs by downregulating ET-1.
